# A New Instrument to Assess Children’s Understanding of Death: Psychometrical Properties of the EsCoMu Scale in a Sample of Spanish Children

**DOI:** 10.3390/children8020125

**Published:** 2021-02-09

**Authors:** Manuel Fernández-Alcántara, Macarena de los Santos-Roig, María Nieves Pérez-Marfil, Francisco Cruz-Quintana, Juan Manuel Vázquez-Sánchez, Rafael Montoya-Juárez

**Affiliations:** 1 Department of Health Psychology, University of Alicante, 03690 Alicante, Spain; mfernandeza@ua.es; 2 Mind, Brain and Behavior Research Center (CIMCYC), University of Granada, 18071 Granada, Spain; dlsantos@ugr.es (M.d.l.S.-R.); fcruz@ugr.es (F.C.-Q.); rmontoya@ugr.es (R.M.-J.); 3 Niño Jesús Hospital, 28009 Madrid, Spain; juanmvazquezsanchez@gmail.com; 4 Faculty of Health Sciences, University of Granada, 18016 Granada, Spain

**Keywords:** death concept, school, causality, irreversibility, universality, non-functionality, children, scale development, neurodevelopment, grief

## Abstract

The acquisition of the death concept in children may influence how these children cope with the losses that they will confront throughout their lives. At the present time, there is a lack of psychometric instruments in Spanish-speaking countries in order to evaluate the components of the death concept in children. The aim of this study was to create and validate a scale (EsCoMu-Escala sobre el Concepto de Muerte) in order to provide insight about the concept of death in children. The sample was formed by 358 children from ages 6 to 13 years. The final EsCoMu version has 27 items which serve to evaluate universality, irreversibility, non-functionality and causality. The results of the confirmatory factor analysis show an adequate fit index for the four dimensions model, reliability (α = 83) and validity evidence, specifically based on the children’s age. In conclusion, EsCoMu is an instrument that shows adequate reliability and validity indices in order to assess the concept of death and its four components among children. Due to its simplicity, this instrument can be very useful if applied to the field of neurodevelopmental disorders.

## 1. Introduction

In Western societies, death is often considered a taboo subject. In the case of children, in addition to the social reticence to talk about death, the way that the concepts of death and dying are learned is extremely important [1] and may influence how they cope with grief and loss. Previous research seems to indicate that children grief manifestations are directly associated to the knowledge they have about death [2]. Also, the possibility to talk about death and understand its meaning may help children to overcome the mistaken ideas or the appearance of unnecessary fears that can have an impact on children’s emotional life even in adulthood, interfering in the normal elaboration of bereavement processes that they will have to deal with in the future [3]. Recent reviews also consider the concept of death as a core aspect when communicating bad news to children and adolescents [4]. The concept of death is very complex, as it is influenced by variables such as social beliefs, cultural norms, emotions, biological development, cognitions or previous experiences with death [5,6]. 

Several studies highlight four core components related to the concept of death: universality, irreversibility, non-functionality and causality [7]. Universality implies that death is conceptualized as a natural phenomenon that applies to all living beings. Irreversibility is linked to the understanding that the dead cannot come back to life. Non-functionality includes the acknowledgement that, once a person has died, their bodily functions cease, as well as their internal and external actions. Lastly, causality implies the understanding of the possible internal or external factors which can cause the end of life. Other authors have proposed other dimensions, such as inevitability, personal mortality or unpredictability [8], but most of them agree on the model proposed by Speece and Brent [7].

There are multiple factors that can influence the understanding of the death concept, even if the scientific evidence of previous investigations is contradictory. Age is one of the most consistent variables, as the death concept is more defined the older the child is. However, each component (universality, irreversibility, non-functionality and causality) seems to follow different patterns [9,10,11,12]. The child’s previous experience with death or illness has also been positively linked to the understanding of the components of the death concept [9]. However, other studies did not find any differences [3,10,13]. The cognitive ability of the children has also been considered an important aspect, especially in the background of the cognitive development model by Jean Piaget. During the preoperational stage, there is a predominance of magical thinking and egocentricity, so it is harder for the child to understand the different aspects of the concept of death. However, De la Herrán et al. [14,15] noted that, as early as 3 years old, children are able to distinguish between life and absence of it. Moreover, between 3 and 5 years old, children start to be curious about the signs of devitalization and the causes of death [16,17]. In subsequent stages, such as in the concrete-operational (7–11 years), the child begin to understand the logical operations and reversibility of thought [18], so they can develop a more mature understanding of death by including components such as irreversibility, non-functionality and causality [19]. Different studies have also indicated the existence of two complementary approaches to death in children that include the biological aspect and the meta-psychological, afterlife or religious conception of death [20]. Both conceptions of death seem to be influenced by culture [21,22].

Keeping in mind the construct complexity, it is necessary to have valid and reliable assessment instruments in order to evaluate the concept of death in children. Previous investigations have used open qualitative interviews, as well as other art-related approaches, such as drawings, storytelling or play in order to investigate the understanding of the death concept [3,9,23]. However, there is a lack of quantitative instruments showing adequate reliability and validity indices in order to assess the concept of death, as, to our knowledge, a Spanish adapted quantitative scale that meets such conditions has yet to be created.

One of the classic resources is the Death Concept Questionnaire [24], which is formed by two groups of 13 questions about death in people and animals. The factor analysis displayed four main factors: irreversibility, non-functionality, causality and inevitability of death and old age [9]. The rating of each item depends on the correctness of the reply (ranging from 0 to 3) and the sophistication of the child’s explanation, finding an overall Cronbach’s alpha of 0.77 [24]. The main limitations of the Death Concept Questionnaire are that each factor is composed of a small number of items and that the responses of the child need to be categorized. 

In Spanish, we can find two qualitative instruments. First, we can find the interview developed by Viñas et al. [25,26], named “Entrevista Estructurada del Concepto de Muerte—ECM” (“Structured Interview on Death Concept”), which evaluates the universality, irreversibility and non-functionality through 11 closed questions with dichotomous answers. Another series of 17 questions are included in order to assess beliefs related to afterlife and the child’s personal experience with death, as well as three open questions where the child is asked to specify three causes of death (animal, person and own) and a final question about the definition of suicide [26]. In Mexico, Gutiérrez et al. [27] have recently developed a qualitative interview in Spanish, where 14 items assessing universality, finality, non-functionality and causality where used. The main limitation of both instruments is that they don’t report evidence involving instrument validity, reliability or factorial structure.

In accordance with the above, it is essential to develop a new instrument in the Spanish language which allows us to assess the components of the death concept in children while having proper psychometric features. The main benefit of having an instrument of this kind is that it can be easily completed and quickly distributed among a large number of participants, without having to invest too much time codifying or correcting the results (as in interviews or other qualitative approaches). Also, the scores from the scale can be easily compared between studies and populations. Finally, we need to establish a reliable measure of the death concept among primary school children which will serve as a starting point to assess other populations of children, such as those with intellectual disabilities or other neurodevelopmental disorders.

Due to this, the main aim of this study is to develop and present the psychometric properties (factorial structure, reliability and validity) of a scale which is able to assess the acquisition of the components of the concept of death in primary school children (6–13 years). The factorial structure of the scale will be tested through confirmatory factor analysis, and reliability will be calculated through internal consistency analysis. The main validity evidence was assessed through the comparison of the scale between different age groups. We hypothesized that younger children will have lower scores in the four dimension of the death concept, in comparison with older children. In addition, we wanted to explore the differences in the following variables: sex, existence of a previous loss and school setting.

## 2. Materials and Methods

The study was formed by 358 primary school students (6–13 years) coming from five schools of the Spanish provinces of Granada, Jaén and Cádiz (see Table 1).

The mean age of children was 9.92 years (SD = 1.57). Different schools of the provinces mentioned before were contacted to select the participant sample. The inclusion criteria were: school’s willingness to participate, informed consent signed by children’s parents or legal guardians, and children who were between 6 and 13 years old. Data were collected about each student’s sex, age and their school setting. Given the foreseeable differences between children of different ages, they were grouped into four levels (see Table 2).

### 2.1. Instruments

Ad-hoc demographic data questionnaire:

Age, sex and school setting (rural/urban/semi-urban) were considered. When the school setting was not fully rural or urban (e.g., towns located in a range of less than 10 km from the province capital), it was coded as semi-urban. A question asking whether they have suffered a recent loss (yes/no) was also added.

Scale to assess the concept of death—EsCoMu (Escala sobre el Concepto de Muerte):

This dichotomous scale was developed by four of the authors of this study who are professionals and researchers specialized on the field of bereavement and end of life processes. The dichotomous rating (yes/no) was based on previous instruments and interviews used in the field [25,28]. The items were evaluated by a panel of experts, who established items relevancy, adequacy and belonging to each of the four dimensions or theoretical components: irreversibility, universality, non-functionality and causality [29]. Each item was rated on a likert scale ranging from 0 to 100 assessing its relevancy (if the item was significantly relevant for the dimension assessed) and adequacy (if the item was appropriate for the proposed dimension of the concept of death). Experts could also include qualitative commentaries about the items. The initial version of the scale consisted on 38 items, of which 10 were eliminated as they showed mean values less of than 70 in any of the dimensions assessed (relevancy and/or adequacy), as well as those that experts identified as difficult to understand for children (in the qualitative part of the survey). An additional item was removed because of their lower factor loadings in the exploratory analysis.

The definitive EsCoMu version is formed by a group of 27 dichotomous items (yes/no answer), grouped into four dimensions: 6 for universality, 7 for irreversibility, 7 for non-functionality and 7 for causality (see Supplementary Material for the Spanish version of the scale). Each item has a score of 1 (if the answer is correct) or 0 (if the answer is incorrect), with some items being reversed. The total score is calculated by adding all items from each component. The scale global Cronbach’s alpha of this investigation was α = 0.83.

### 2.2. Procedure

Two informative meetings were held in order to inform the management of each school participating in the study. After obtaining the school’s permission, with the help of Parent Associations (AMPA—Asociación de Madres y Padres de Alumnos), an informative meeting with parents was held in order to explain the aim of the study. Secondly, each student was given an informative sheet and an informed consent in order to be signed by their parents. Subsequently, this consent was returned to the school teacher along with each parent’s or legal guardian’s signature. The students whose parents did not fill in the informed consent performed a different activity unrelated to the study.

The assessment was performed in groups, in the students’ regular classroom. In one session, they filled in all the sociodemographic data as well as the scale EsCoMu. The evaluation lasted around 20 min, and all students received similar guidelines. The evaluation was performed by a team of experts experienced in end-of-life processes.

At the beginning of the evaluation, all students were given the option of not participating in the activity if they did not want to, regardless of the parent’s consent. Each participant’s emotional state was evaluated during and after the assessment in order to provide them with emotional support if needed, but none of the participants had reported issues in this regard.

### 2.3. Ethical Considerations

Prior to the data collection and the inclusion of participants in the study, both school management and parents were informed about the aim, purpose and confidentiality of the study. In every case, the evaluation was performed after obtaining both the school authorization and after collecting parent-signed informed consent for their children’s participation. The present study was approved by the University of Granada’s Ethics Committee on Human Research (Ref. 1056/CEIH/2020).

### 2.4. Data Analysis

To perform the descriptive analysis, the frequency of each answer was calculated for every item. To verify the factor structure, a confirmatory factor analysis (CFA) was performed. Given the dichotomous condition of data, the WLSMV (Variance-Adjusted Weighted Least Squares) estimation method was used. The following indices were used in order to calculate items’ fit with the proposed model: RMSEA (Root Mean Square Error of Approximation), TLI (Tucker–Lewis Index), CFI (Comparative Fit Index) and WRMR (Weighted Root Mean Square Residual).

To gather validity evidence about possible relations between EsCoMu’s ratings and other external variables, multivariate analysis of variance (MANOVA) was applied, with variables including age, sex, having suffered a loss (yes/no) and school setting (urban, semi-urban and rural) for each subcomponent of the scale. For post-hoc contrasts, Bonferroni correction and partial eta-squared effect size were used. Statistical software SPSS 22 (IBM, 2013) was used. For gathering structural validity evidence, MPLUS 6.11 [30] was used.

## 3. Results

### 3.1. Descriptive Analysis of EsCoMu’s Response

In Table 3, the percentage of correct answers for each age group is shown in each EsCoMu scale item, based on their dimension.

### 3.2. EsCoMu Factor Structure

**Figure 1:**
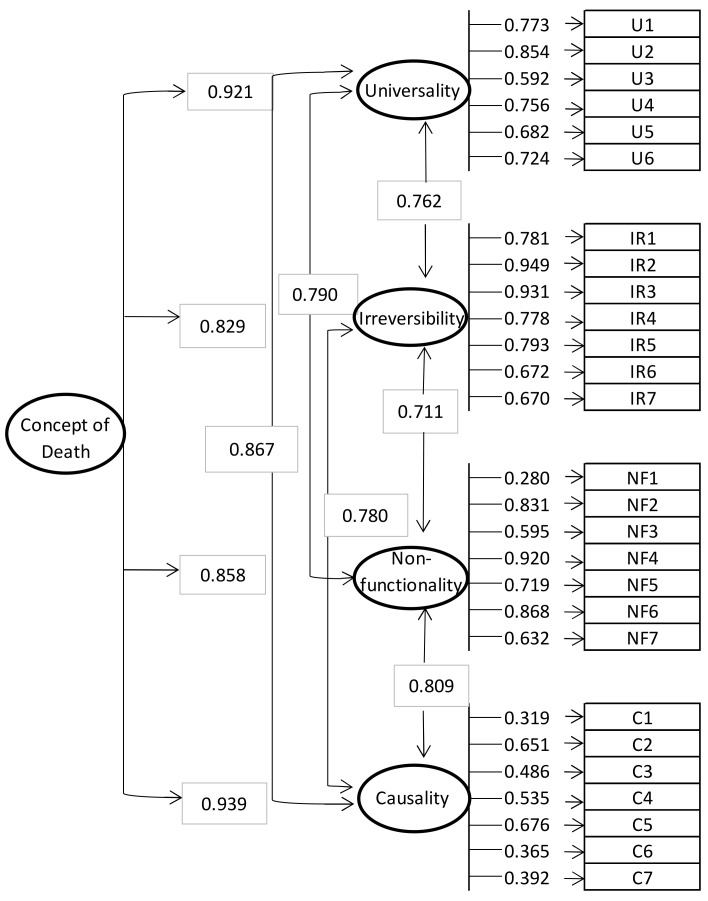
Second-order four factors EsCoMu model. Latent variables are represented by ellipses and measured variables are represented by rectangles. Values are standardized estimated correlations between factors.

Two one-order models (One-Factor and Four-Factor) were tested. However, fit indices for the one-factor model (see Table 4) were not adequate. As correlation values were not appropriate for causality-universality dimensions on the Four-Factor model (with a coefficient bigger than 1), a second-order model was tested (Second order with Four Factor model). CFA results showed adequate fit indices in this model (see Table 4 and Figure 1). The dimensions showed medium-high values of intercorrelation (see Table 5).

### 3.3. Evidence of Validity

As observed in Table 6, the MANOVA results indicate a significative medium-low effect size with age in all EsCoMu dimensions as well as in the total score. Post-hoc analysis (Bonferroni) indicate lower scores in Universality, Irreversibility, and Non-functionality in the 6–7 years age group if compared with the 8–9 years age group (*p* < 0.01) and with the 10–11 years age group (*p* < 0.01). There were no statistically significant differences among the youngest and oldest age groups. However, both in the Causality dimension and in the total score, the youngest children showed statistically significant differences (both *p* < 0.01) if compared with other groups, where such differences do not occur.

As observed in Table 7 and Table 8, there were no differences between any of the instrument dimensions involving sex, nor between the participants who have suffered a recent loss and those who did not.

Finally, when analyzing the children’s school setting, MANOVA showed lower results in rural areas (Table 9). The effects appear in the four dimensions as well as in the instrument’s total score, with medium-to-low effect sizes. The post-hoc analysis results (Bonferroni) indicate differences only between rural and semi-urban settings (*p* < 0.01) in terms of Universality, Irreversibility and Non-functionality. Again, in this case, Causality and the EsCoMu total score are the dimensions with more differences between rural settings and the other two groups (*p* < 0.05).

## 4. Discussion

The aim of this study was to develop and present the psychometric properties (factor structure, reliability and validity) of a scale which was able to assess the acquisition of the components of the concept of death in primary school children (6–13 years). Results prove that the scale has an adequate reliability and factor structure, showing promising validity evidences.

The scale global alpha shows acceptable values, in the same direction as the value reported to this date by the Death Concept Questionnaire (α = 0.77 in the original study and α = 0.81 in the study by Bonoti et al. [9]). Test–retest measures could not be included, so future research should investigate whether the EsCoMu scale maintains its reliability over time, as well as whether it is sensitive to death education-based interventions [2,14].

The four scale factors showed positive and moderate correlations between each other. Furthermore, the CFA model showed that the four components of the concept are closely related to each other, suggesting that they are part of an underlying construct, in this case the concept of death. This is supported by the results of the second-order CFA, where the concept of death is explained by the four factors, which are also explained by their respective items. Following this rationale, previous research has highlighted the relevance of these four components to explain the concept of death [6,10].

Age is one of the factors that seems to systematically influence the acquisition of the concept of death. In the present study, age-dependent differences were found in the four evaluated dimensions, in line with previous investigations [10,31]. In the dimensions of universality, irreversibility and non-functionality, differences were identified between younger and older children, whereas, in causality, statistically significant differences were found between 6–7-year-old children and the rest of the age groups. This seems to indicate that not all dimensions are acquired by following the same pattern. However, we do observe that, from the age of 8–9 years, all four subcomponents are well acquired in children [7,9]. In their sample, Gutiérrez et al. [27] found that not all the components of the death concept differed according to age, finding no significant differences in universality and causality based of this variable, but rather between finality and non-functionality. However, results for these variables were very close to significance (*p* < 0.08 in both cases).

Regarding the school setting, lower scores have been found in rural schools as compared to these semi-urban and urban schools. Previous studies have shown different results, pointing to greater acquisition in children living in rural environments. Panagiotaki et al. [31] found significant differences in the irreversibility component when comparing three children groups (British living in London, British Muslims living in London, and Muslims living in rural areas of Pakistan), being higher in the latter group. However, in this study, since the groups were not equivalent due to a series of key cultural variables, no conclusive evidence can be drawn regarding the area of origin. Lastly, other studies did not find significative differences regarding the death concept in urban and rural settings [32].

In the present study, we also did not find significant differences in any of the components of the death concept based on the child’s sex or in the event of having a recent loss. These findings are consistent with one prior study [3], but differ from another study that found differences on this variable [9]. Future studies must investigate the influence of the loss type and the appearance of specific symptoms in terms of bereavement, which sheds light on to what extent it is the experience of loss itself, or the intensity of the child’s experience that will affect the acquisition of the concept of death [33].

The development of a scale that serves to evaluate the concept of death has important clinical implications. On one hand, death is not a common topic in school subjects or academic curricula. On the other hand, adults and families are often hesitant about how to respond to the questions that children raise about the concept of death and the dying process. Moreover, in spite of children’s curiosity, some adults are unsure about the appropriateness of discussing death with their children [34]. Therefore, it is a common situation that children are not able to find the adequate space to clarify their doubts regarding what death means. This may prevent them from developing more adaptive coping responses, which can lead to emotional issues. Therefore, it is essential to have valid and reliable instruments which allow us to evaluate the conceptualization of death in different ages and contexts, as well as to work in education, as many teachers are currently demanding [35,36].

The EsCoMu scale, due to its fast and easy application, can be widely used in populations with neurodevelopmental disorders or problems. Previous qualitative studies have shown that, in cases involving diagnosed intellectual disabilities or neurodevelopmental conditions, the acquisition of the death concept seems to follow a different pattern. Markell and Hoover [37] highlight how even learning problems or physical and emotional issues in children can affect the bereavement process and understanding of death. Children diagnosed with intellectual disability (ID) have shown confusion and difficulty understanding the concepts of non-functionality, irreversibility (associating death with the illness) and universality [37]. Finally, recent studies in adults diagnosed with ID show that they do acquire the components of the death concept, but in a different and, in many cases, partial way [38,39], showing greater difficulty understanding concepts such as causality and universality [40]. Future studies that use the EsCoMu scale may explore the concept of death in these populations, as well as the effectiveness of interventions related to death education and supporting methods for bereavement and end-of-life processes in this population [41].

However, this research has a series of limitations: Firstly, the child age groups were not equivalent, the 10–11-year-old group being the biggest one. Moreover, it is necessary for future research to examine the usefulness of EsCoMu among populations under 6 years of age as well as to include test–retest measures to evaluate the temporal reliability of the scale. We did not perform a pilot study or cognitive interviews prior to the initial assessment, so it may be useful to perform pilot testing when applying this scale to children less than 6 years old. However, the assessed population did not have any problem understanding any of the items. No measures of anxiety or depression were taken in the children who completed the scale, so it would be necessary in the future to control such variables and check their effect and connection to the EsCoMu score. Future studies should verify if the acquisition of the components of the death concept correlates with the most common themes regarding death, such as biological, psychological or metaphysical death [3,9]. In the present study, age was considered as the main evidence of validity, but there are other variables associated with the concept of death, such as religious, cognitive or socioeconomic aspects, that should be included in future studies to have measures of convergent validity of the EsCoMu scale. Finally, the use of mixed methods design to explore the relationship between the acquisition of the components of the concept of death and the subjective experience of the child [27] will give additional information about the validity of the EsCoMu scale.

## 5. Conclusions

In conclusion, the EsCoMu scale is an instrument with adequate factor structure that shows adequate reliability and validity indices in order to assess the concept of death and its four components (universality, irreversibility, non-functionality and causality) among children.

## Figures and Tables

**Table 1:** Schools in which the sample is taken.

Town	Province	Setting	Management	Religious	Participants
La Zubia	Granada	Semi-urban	Public	No	105
Granada	Granada	Urban	Semi-private	Yes	83
Arjonilla	Jaén	Rural	Public	No	81
Chiclana	Cádiz	Urban	Public	No	44
Chiclana	Cádiz	Urban	Public	No	45

**Table 2:** Sociodemographic data sample divided by age group.

		Groups			
		6–7 Years	8–9 Years	10–11 Years	12–13 Years
	*n* (%)	34 (9.5%)	96 (26.9%)	189 (52.9%)	38 (10.6%)
Sex	Female	17 (50%)	56 (58.3%)	95 (50.5%)	16 (43.2%)
	Male	17 (50%)	40 (41.7%)	93 (49.5%)	21 (56.8%)
School Setting	Rural	18 (52.9%)	19 (33.9%)	24 (12.8%)	11 (29.7%)
	Semi-urban	0	22 (26.8%)	74 (39.4%)	0
	Urban	16 (47.1%)	15 (40.5%)	90 (47.9%)	26 (70.3%)
Recent Loss	Yes	21 (61.8%)	63 (65.6%)	118 (62.45%)	22 (57.9%)
	No	13 (38.2%)	33 (34.4%)	71 (37.6%)	16 (42.18%)

**Table 3:** Descriptive analysis of the EsCoMu scale items for each age group.

		Correct Answer (%)			
Item	Content	6–7 Years (*n* = 34)	8–9 Years (*n* = 96)	10–11 Years (*n* = 198)	12–13 Years (*n* = 38)
U1	Do you think that your grandparents will die someday?	85.3	92.7	96.8	81.6
U2 *	Can a mum live forever?	88.2	93.7	98.4	81.0
U3	Can you die?	81.3	93.7	94.7	86.8
U4 *	Are there living beings that do not die?	69.7	88.5	90.4	84.2
U5	Do all people die?	79.4	89.6	98.4	92.1
U6	Do you think that a very good person can die?	64.7	86.2	95.8	86.5
IR1 *	If we die, can someone wake us up?	76.5	84.2	96.8	86.8
IR2 *	Can a dead animal come back to life?	72.7	94.6	96.3	86.8
IR3 *	If someone close to you died, could they come back to life?	78.8	88.4	93.0	83.8
IR4 *	If a child died, could they live again?	73.5	91.5	92.6	78.9
IR5 *	Can a dead person come back to life if you really want it?	85.3	90.4	96.3	91.9
IR6 *	Can a dead person come back to life?	82.4	96.2	87.7	92.1
IR7 *	After dying, do you think that it is possible to come back to life?	85.3	87.1	86.1	78.9
NF1 *	When someone dies, can they feel cold?	81.8	74.0	72.6	65.8
NF2 *	Do animals feel hunger or thirst when they are dead?	82.4	90.5	96.3	92.1
NF3 *	Can a dead person move?	70.6	89.6	93.1	97.4
NF4 *	Does a dead person keep breathing?	87.9	93.7	98.4	94.7
NF5	When someone dies, does their body stop working?	70.6	89.2	91.0	92.1
NF6 *	When an animal dies, do they still want to play?	75.8	91.6	96.8	81.6
NF7 *	Can a dead person hear or feel?	76.5	81.9	87.2	91.9
C1	Can a person kill themselves?	79.4	93.6	98.9	86.8
C2	Can people die of hunger or thirst?	67.6	85.1	87.8	86.5
C3	Can a person die from falling off a high place?	91.2	94.8	97.4	97.2
C4 *	Can someone be killed by the force of imagination?	68.8	86.3	84.5	83.8
C5	Can a person die from being very old?	81.8	86.5	97.3	91.9
C6	Can people die after suffering an illness for a long time?	93.9	98.9	98.9	97.3
C7	Can a person die if they have a serious accident?	97.1	97.9	96.8	94.7

* Reverse items, U = Universality, IR = Irreversibility, NF = Non-Functionality, C = Causality.

**Table 4:** Fit indices for EsCoMu factor models.

Model	Χ^2^	*df*	CFI/TLI	RMSEA	WRMR
One factor	449.86 **	324	0.885/0.876	0.033 [C.I. _95%_ = 0.025–0.040]	1.071
Four factors	398.08 **	318	0.927/0.919	0.027 [C.I. _95%_ = 0.017–0.035]	0.951
Second-order four factors	406.37 **	320	0.921/.914	0.028 [C.I. _95%_ = 0.018–0.035]	0.971

Note. ** = *p* < 0.001, Χ^2^ = Chi Square goodness of fit statistic, *df* = degrees of freedom, CFI = Comparative Fit Index, TLI = Tucker–Lewis Index, RMSEA = Root Mean Square Error of Approximation, WRMR = Weighted Root Mean Square.

**Table 5:** Intercorrelations between EsCoMu dimensions.

	Irreversibility	Non-Functionality	Causality
1. Universality	0.431 **	0.363 **	0.552 **
2. Irreversibility		0.424 **	0.376 **
3. Non-functionality			0.312 **

Note. ** = *p* < 0.01.

**Table 6:** MANOVA results for school setting with EsCoMu dimensions and total scores.

EsCoMu Dimension	Age (*n*)	Mean (SD)	F (df)	Power	η^2^
1	6–7 (24)	4.79 (1.35)	F(_3,303_) = 9.65 **	0.99	0.08
	8–9 (75)	5.65 (0.84)			
	10–11 (177)	5.74 (0.63)			
	12–13 (31)	5.35 (1.35)			
2	6–7 (24)	5.37 (2.22)	F(_3,303_) = 5.29 **	0.92	0.05
	8–9 (75)	6.44 (0.87)			
	10–11 (177)	6.45 (1.20)			
	12–13 (31)	6.12 (1.70)			
3	6–7 (24)	6.20 (2.08)	F(_3,303_) = 4.14 **	0.84	0.04
	8–9 (75)	7.14 (1.15)			
	10–11 (177)	7.22 (1.24)			
	12–13 (31)	7.00 (1.57)			
4	6–7 (24)	5.62 (1.05)	F(_3,303_) = 10.82 **	0.99	0.09
	8–9 (75)	6.53 (0.74)			
	10–11 (177)	6.61 (0.64)			
	12–13 (31)	6.38 (1.35)			
Total Score	6–7 (24)	21.25 (5.30)	F(_3,303_) = 12.11 **	1.00	0.10
	8–9 (75)	24.90(2.24)			
	10–11 (177)	25.14 (2.40)			
	12–13 (31)	24.09 (5.00)			

Note. 1. = Universality, 2. = Irreversibility, 3. = Non-functionality, 4. = Causality, SD = Standard Deviation, df = degree of freedom, η^2^ = partial eta-squared effect size, ** = *p* < 0.01.

**Table 7:** MANOVA results for gender with EsCoMu dimensions and total scores.

EsCoMu Dimension	Sex (*n*)	Mean (SD)	F (df)	Power	η^2^
1	Male (150)	5.58 (0.80)	F(_1,304_) = 0.28	---	---
	Female (156)	5.63 (0.97)			
2	Male (150)	6.35 (1.36)	F(_1,304_) = 0.06	---	---
	Female (156)	6.31 (1.30)			
3	Male (150)	7.18 (1.25)	F(_1,304_) = 0.90	---	---
	Female (156)	7.03 (1.45)			
4	Male (150)	6.54 (0.69)	F(_1,304_) = 1.02	---	---
	Female (156)	6.44 (0.96)			
Total Score	Male (150)	24.76 (2.92)	F(_1,304_) = 0.23	---	---
	Female (156)	24.58 (3.44)			

Note. 1. = Universality, 2. = Irreversibility, 3. = Non-functionality, 4. = Causality, SD = Standard Deviation, df = degree of freedom, η^2^ = partial eta-squared effect size

**Table 8:** MANOVA results for recent loss with EsCoMu dimensions and total scores.

EsCoMu Dimension	Loss (*n*)	Mean (SD)	F (df)	Power	η^2^
1	Yes (198)	5.60 (0.90)	F(_1,306_) = 0.001	--	--
	No (110)	5.61 (0.86)			
2	Yes (198)	6.33 (1.31)	F(_1,306_) = 0.034	--	--
	No (110)	6.30 (1.36)			
3	Yes (198)	7.05 (1.42)	F(_1,306_) = 0.363	--	--
	No (110)	7.15 (1.30)			
4	Yes (198)	6.548 (0.86)	F(_1,306_) = 0.001	--	--
	No (110)	6.49 (0.79)			
Total Score	Yes (198)	24.61 (3.20)	F(_1,306_) = 0.07	--	--
	No (110)	24.71 (3.23)			

Note. 1. = Universality, 2. = Irreversibility, 3. = Non-functionality, 4. = Causality, SD = Standard Deviation, df = degree of freedom, η^2^ = partial eta-squared effect size

**Table 9:** MANOVA results for school setting with EsCoMu dimensions scores.

EsCoMu Dimension	School Setting (*n*)	Mean (SD)	F (df)	Power	η^2^
1	Urban (135)	5.57 (0.95)	F(_2,305_) = 5.67 *	0.86	0.03
	Rural (77)	5.38 (1.10)			
	Semi-urban (96)	5.83 (.45)			
2	Urban (135)	6.32 (1.37)	F(_2,305_) = 7.64 **	0.94	0.05
	Rural (77)	5.89 (1.63)			
	Semi-urban (96)	6.67 (.81)			
3	Urban (135)	7.70 (1.41)	F(_2,305_) = 4.60 *	0.77	0.03
	Rural (77)	6.75 (1.58)			
	Semi-urban (96)	7.38 (1.07)			
4	Urban (135)	6.57 (.85)	F(_2,305_) = 8.02 **	0.95	0.05
	Rural (77)	6.16 (.95)			
	Semi-urban (96)	6.63 (.63)			
Total Score	Urban (135)	24.70 (3.46)	F(_2,305_) = 10.24 **	0.98	0.06
	Rural (77)	23.41 (3.82)			
	Semi-urban (96)	25.57 (1.59)			

Note. 1. = Universality, 2. = Irreversibility, 3. = Non-functionality, 4. = Causality, SD = Standard Deviation, df = degree of freedom, η^2^ = partial eta-squared effect size, * = *p* < 0.05, ** = *p* < 0.01.

## Data Availability

Data will be available upon request to the corresponding author.

## Supplementary Materials

The following are available online at https://www.mdpi.com/2227-9067/8/2/125/s1, Table S1: Escala sobre el Concepto de Muerte (EsCoMu).

## References

[1] Pla A.R., Guàrdia R.C. (2018). Fundamentos para una pedagogía preventiva sobre la muerte en la escuela. Rev. Complut. Educ..

[2] Serrano-Pastor F.J., Martínez-Segura M.J. (2019). Las Cosas Que No Me Cuentas. Propuesta de Innovación Educativa para la Pérdida y el Duelo.

[3] Vázquez-Sánchez J.M., Fernández-Alcántara M., García-Caro M.P., Cabañero-Martínez M.J., Martí-García C., Montoya-Juárez R. (2019). The concept of death in children aged from 9 to 11 years: Evidence through inductive and deductive analysis of drawings. Death Stud..

[4] Stein A., Dalton L., Rapa E., Bluebond-Langner M., Hanington L., Fredman-Stein K., Ziebland S., Rochat T., Harrop E., Kelly B. (2019). Communication with children and adolescents about the diagnosis of their own life-threatening condition. Lancet.

[5] Krepia M., Krepia V., Tsilingiri M. (2017). School children’s perception of the concept of death. Int. J. Caring Sci..

[6] Kronaizl S.G. (2019). Discussing death with children: A developmental approach. Pediatr. Nurs..

[7] Speece M.W., Brent S.B. (1984). Children’s understanding of death: A review of three components of a death concept. Child Dev..

[8] Slaughter V. (2005). Young children’s understanding of death. Aust. Psychol..

[9] Bonoti F., Leondari A., Mastora A. (2013). Exploring children’s understanding of death: Through drawings and the death concept questionnaire. Death Stud..

[10] Panagiotaki G., Hopkins M., Nobes G., Ward E., Griffiths D. (2018). Children’s and adults’ understanding of death: Cognitive, parental, and experiential influences. J. Exp. Child Psychol..

[11] Longbottom S., Slaughter V. (2018). Sources of children’s knowledge about death and dying. Philos. Trans. R. Soc. B Biol. Sci..

[12] Norero V. (2018). La maduración cerebral en el niño. El caso de la adquisición del concepto de muerte y su evolución. Rev. Chil. Pediatr..

[13] Miller P.J., Rosengren K.S., Gutiérrez I.T. (2014). Children’s understanding of death: Toward a contextualized and integrated account: I. Introduction. Monogr. Soc. Res. Child Dev..

[14] De la Herrán A., Cortina M. (2009). La muerte y su enseñanza. Diálogo Filosófico.

[15] De la Herrán A., Herrero P.R., de Miguel Yubero V. (2019). Is death in the Spanish curriculum?. Rev. Educ..

[16] Tau R., Lenzi A. (2012). Acerca del desarrollo de la noción de muerte en niños. Anu. Investig. Fac. Psicol..

[17] Mahmood Ashiri R., Khodabakhshi-Koolaee A. (2020). Explaining the concept of death from the perspective of children aged 4 to 8: A descriptive phenomenological study. J. Qual. Res. Health Sci..

[18] Piaget J. (1963). The Origins of Intelligence in Children.

[19] Paul S. (2019). Is Death Taboo for Children? Developing Death Ambivalence as a Theoretical Framework to Understand Children’s Relationship with Death, Dying and Bereavement. Child. Soc..

[20] Harris P.L. (2018). Children’s understanding of death: From biology to religion. Philos. Trans. R. Soc. B Biol. Sci..

[21] Giménez M., Harris P. (2005). Children’s acceptance of conflicting testimony: The case of death. J. Cogn. Cult..

[22] Astuti R., Harris P.L. (2008). Understanding mortality and the life of the ancestors in rural Madagascar. Cogn. Sci..

[23] Yang S., Park S. (2017). A sociocultural approach to children’s perceptions of death and loss. Omega J. Death Dying.

[24] Smilansky S. (1987). On Death: Helping Children Understand and Cope.

[25] Viñas i Poch F., Jané i Ballabriga M., Domènech E. (2000). Assessment of self-report suicidal ideation severity in 8 to 12 years old school children. Psicothema.

[26] Viñas i Poch F., Domènech E. (1999). El concepto de muerte en un grupo de escolares con ideación suicida. Rev. Psicol. Gen. Appl..

[27] Gutiérrez I.T., Menendez D., Jiang M.J., Hernandez I.G., Miller P., Rosengren K.S. (2020). Embracing death: Mexican parent and child perspectives on death. Child Dev..

[28] Speece M.W., Brent S.B. (1992). The acquisition of a mature understanding of three components of the concept of death. Death Stud..

[29] Fernández-Alcántara M., Cruz-Quintana F., Pérez-Marfil M.N., Saforcada E., Fariña O. (2017). Mecanismos emocionales y neuropsicológicos en procesos de duelo complicado. Neurociencias Aplicadas. Medioambiente, Desarrollo Humano y Bienestar Comunitario.

[30] Muthén L.K., Muthén B.O. (2007). Mplus User’s Guide.

[31] Panagiotaki G., Nobes G., Ashraf A., Aubby H. (2015). British and Pakistani children’s understanding of death: Cultural and developmental influences. Br. J. Dev. Psychol..

[32] Hopkins M. (2014). The Development of Children’s Understanding of Death. Ph.D. Thesis.

[33] Speece M.W. (1995). Children’s concepts of death. Mich. Fam. Rev..

[34] Flahault C., Dolbeault S., Sankey C., Fasse L. (2018). Understanding grief in children who have lost a parent with cancer: How do they give meaning to this experience? Results of an interpretative phenomenological analysis. Death Stud..

[35] Siracusa F., Cruz-Quintana F., Perez-Marfil M.N., Garcia-Caro M.P., Schmidt-Riovalle J., Vera-Martinez M. (2011). Actitudes y afrontamiento ante la muerte en padres de niños de primaria. Psicol. Conduct..

[36] Morell-Velasco C., Fernández-Alcántara M., Hueso-Montoro C., Montoya-Juárez R. (2020). Teachers’ Perception of Grief in Primary and Secondary School Students in Spain: Children’s Responses and Elements Which Facilitate or Hinder the Grieving Process. J. Pediatr. Nurs..

[37] Markell M.A., Hoover J.H., Corr C.A., Balk D.E. (2010). Children with developmental disabilities, death, and grief. Children’s Encounters with Death, Bereavement, and Coping.

[38] McEvoy J., MacHale R., Tierney E. (2012). Concept of death and perceptions of bereavement in adults with intellectual disabilities. J. Intellect. Disabil. Res..

[39] McEvoy J., Treacy B., Quigley J. (2017). A matter of life and death: Knowledge about the body and concept of death in adults with intellectual disabilities. J. Intellect. Disabil. Res..

[40] Chow A.Y.M., McEvoy J., Chan I.K.N., Borschel M., Yuen J.H.L., Lo J.Y.M. (2017). Do men and women with intellectual disabilities understand death?. J. Intellect. Disabil. Res..

[41] Fernández-Ávalos M.I., Pérez-Marfil M.N., Ferrer-Cascales R., Cruz-Quintana F., Fernández-Alcántara M. (2020). Feeling of grief and loss in parental caregivers of adults diagnosed with intellectual disability. J. Appl. Res. Intellect. Disabil..

