# Supplementary material for: A New Instrument to Assess Children’s Understanding of Death: Psychometrical Properties of the EsCoMu Scale in a Sample of Spanish Children
Source: Children (Basel). 2021 Feb 9;8(2):125. doi: 10.3390/children8020125 (PMC7916254; doi:10.3390/children8020125)
Supplement: Supplementary file 1 [file children-08-00125-s001.pdf]

Table S1. Escala sobre el Concepto de Muerte (EsCoMu)

A continuación voy a realizarte algunas preguntas sobre qué crees que ocurre cuando nos morimos. Me gustaría que me contestaras a cada una de ellas respondiendo sí o no.

	**Dimensión 1. Universalidad**	Sí	No
U1	¿Crees que tus abuelos morirán algún día?		
U2*	¿Y una mamá, puede vivir siempre?		
U3	¿Tú te puedes morir?		
U4*	¿Hay seres vivos que nunca se mueren?		
U5	¿Todas las personas se mueren?		
U6	¿Crees que alguien que sea muy bueno puede morir?		
	**Dimensión 2. Irreversibilidad**	Sí	No
IR1*	Si nos morimos, ¿alguien nos puede despertar?		
IR2*	¿Un animal que ha muerto puede volver a vivir?		
IR3*	¿Si alguien cercano a ti muriese podría volver a la vida?		
IR4*	¿Si un niño muriese podría volver a vivir?		
IR5*	¿Alguien que muera puede volver a vivir si lo deseas con todas tus fuerzas?		
IR6*	¿Puede revivir alguien que se ha muerto?		
IR7*	¿Crees que tras morir es posible volver a la vida?		
	**Dimensión 3. Cesación de procesos corporales**	Sí	No
CS1*	Cuando alguien se muere, ¿puede tener frío?		
CS2*	¿Los animales tienen hambre o sed cuando se han muerto?		
CS3*	¿Una persona que ha muerto se puede mover?		
CS4*	¿Alguien que ha muerto sigue respirando?		
CS5	¿Cuando alguien muere su cuerpo deja de funcionar?		
CS6*	¿Cuando un animal muere sigue teniendo ganas de jugar?		
CS7*	¿Una persona que se ha muerto, puede oír o sentir?		

	**Dimensión 4. Causalidad**	Sí	No
CA1	¿Una persona, se puede matar a sí misma?		
CA2	¿Las personas pueden morir de hambre o de sed?		
CA3	¿Alguien que se caiga desde mucha altura puede morir?		
CA4*	¿Se puede matar a alguien con la imaginación?		
CA5	¿Una persona se puede morir por ser ya muy mayor?		
CA6	¿Las personas pueden morirse tras una larga enfermedad?		
CA5	¿Puede una persona morir si tiene un accidente grave?		

Nota. * Ítem inverso
